# Laser-induced melting of two-dimensional dusty plasma system in RF discharge

**DOI:** 10.1038/s41598-020-80082-x

**Published:** 2021-01-12

**Authors:** E. V. Vasilieva, O. F. Petrov, M. M. Vasiliev

**Affiliations:** 1 Joint Institute for High Temperatures, Russian Academy of Sciences, Moscow, Russia; 2 Moscow Institute of Physics and Technology, Dolgoprudny, Russia

**Keywords:** Phase transitions and critical phenomena, Plasma physics

## Abstract

We present a detailed analysis of experimental study, which shows clear evidence of a two-stage melting process of a quasi-two-dimensional dusty plasma system in a high-frequency gas discharge. We accurately calculated global parameters of the orientational and translational order, as well as their susceptibilities to determine two critical points, related to “solid-to-hexatic” and “hexatic-to-liquid” phase transitions. The nature of the emerging defects and changes in their mutual concentration, in addition to the estimate of core energy of free dislocations also counts in favor of the formation of an intermediate hexatic phase. These results are fully consistent with the Berezinsky–Kosterlitz–Thouless theory.

## Introduction

Two-dimensional (2D-) melting is still one of the unresolved entirely problems in condensed and soft matter physics^1–4^, and over the decades two main competing theories, attempting to describe 2D melting, have emphasized main role of topological defects and grain boundaries. The first approach is Berezinsky–Kosterlitz–Thouless (BKT-) theory^5–8^, developed later by B. Halperin, D.R. Nelson and A.P. Young^9,10^ and transformed eventually to Berezinsky–Kosterlitz–Thouless–Halperin–Nelson–Young (BKTHNY-) theory^11^, which predicts two-step melting, from the crystal to liquid phase with formation of intermediate hexatic phase. It has short range translational and quasi-long-range orientational order, and phase transitions are caused by the creation and dissociation, binding and unbinding of topological defects, such as disclinations and dislocations. Alternative theories consider a conventional first-order phase transition from solid to liquid without the formation of any intermediate phase^12,13^, assuming that the condensation of geometrical defects into grain boundaries and related aggregates is responsible for two-dimensional melting.

Researchers have been looking for the evidence of hexatic phase among a wide range of two-dimensional systems^14–45^, including monolayers of electrons on the surface of liquid helium^14^, polymer colloids^15–17^, magnetic bubbles in thin films^18–20^, liquid crystals^21–23^, superconductors^24^, as well as in dusty plasmas^25–45^. Some experimental and numerical works have shown a good agreement with KTHNY theory^25–28^, but others haven't. The reason of that might be in out-of-plane fluctuations^29,30^, finite-size effects^31,32^ or different types of inhomogeneities, commonly observed in experiments during melting process. But the most crucial problem is concerned with the interaction range and stationarity of the system state. It is more complicated to get clear evidence of hexatic phase in systems with short-range interactions (for instance, in dusty plasma systems with grains, interacting via Yukawa potential: φ(*r*) = *eZ* exp(− *r*/λ)/*r*, where λ is a screening length, and *eZ* is a charge of the particles) than with long-range potentials^33–35^. In work^36^ the authors showed that the formation of an intermediate hexatic phase had been strongly dependent on the thermodynamic equilibration of the observed two-dimensional system. More specifically, long-range or collective relaxation had taken much more time in comparison with that one, related to single-particle local properties of the system.

First experimental studies of phase transitions in 2D dusty plasmas have been carried out by changing parameters of gas discharge^37,38^, namely gas pressure or discharge power. That in turn led to a change of plasma parameters and confinement and therefore the interaction potential of dust particles in these structures. Further studies^39,40^ showed that in that case system exhibited non-equilibrium melting due to occurring of mode-coupling instability. Other experimental studies^41–45^ presented different methods, none of which provided reliable degree of uniformity of heating of dusty plasma system as a whole. As the result, the authors concluded that the GBI-theory was best suited to a description of melting of two-dimensional dust structures, observed in their experiments. And finally, in our work^28^ we presented convincing evidence of formation of hexatic phase during 2D melting of dusty plasma system, which was experimentally observed for the first time.

Conventional analysis of any system near the phase transition points is much more complicated due to the growth of various fluctuations arising in this system. Standard approach, such as analysis of order parameter correlation functions, makes it possible to distinguish different phase states, but suffer ambiguities connected with finite size of the system and/or finite time of observation. Conversely, order parameter susceptibility method, presented in^46^ and applied in colloids^17^ showed to be robust to various uncertainties and enabled to clearly resolve phase transition points.

In this work, we introduce extended analysis of structural properties of 2D dusty plasma system, based on structure factor; discuss dynamics of topological defects together with assessment of core energy of free dislocations; and calculate order parameter susceptibility, which enabled us to determine accurately “solid-to-hexatic” and “hexatic-to-liquid” phase transition points.

## Experiments

**Figure 1:**
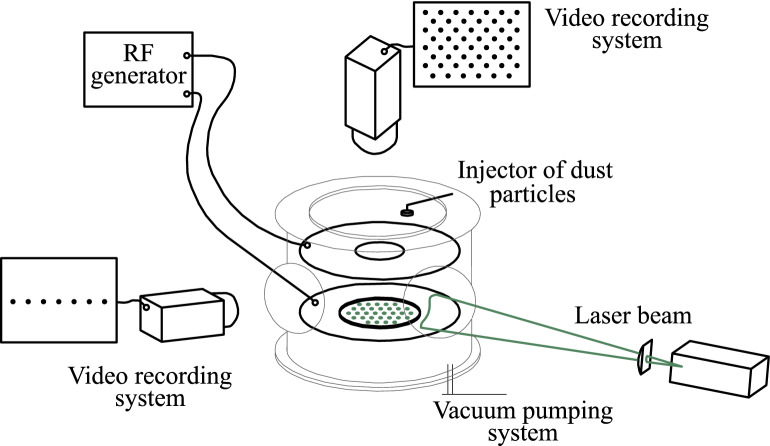
The scheme of the experimental setup.

The experiments were performed in a RF discharge in argon at pressure *P* = 4.3 Pa, with discharge power *W* = 7.9 W. As dust component we used spherical monodisperse polystyrene particles, of density *ρ*_*d*_ = 1.05 g cm^-3^, with diameter 10.16 μm, coated with nickel of 200 nm in thickness. The scheme of the experimental setup is presented in Fig. 1. Main part of the experimental setup was vacuum chamber, which was preliminary pumped out and then filled by buffer gas up to working pressure. RF discharge was generated between two plane circular coaxial electrodes by high-frequency generator of 13.56 MHz with the use of impedance-matching device. The distance between the electrodes was 5 cm. In the center of upper electrode there was a circular hole, which enabled us to record video in horizontal plane as well as to inject particles into discharge gap. After injection dust particles gained equilibrium negative charges because of ion and electron fluxes going to the particle surface until a stationary state is reached. Due to the balance of gravity and electrostatic force, providing a stiff vertical confinement, charged particle levitated above a flat horizontal rf electrode forming monolayer structures with negligible small vertical motions. To prevent the escape of particles in the horizontal direction metal ring with a height of about 2 mm and 100 mm in diameter was mounted on the lower (grounded) electrode; the ring formed a potential trap for the cloud of dust particles.

For visualization and external influence on the dusty plasma structure we used argon laser. The laser power varied in the range from 20 to 300 mW. In contrast to works^40–45^, where uncoated particles were used and external action caused non-uniform heating, we used metal-coated particles with completely different response. Interacting with the metal-coated surface of particles, the laser light warmed them up, and a part of their surface became heated. Colliding with it, the neutrals of a buffer gas induced the photophoretic force^47,48^. As the grains rotated due to the Brownian motion, this force uniformly acted on the whole surface of grains and, therefore, increased the energy of their chaotic motion, so that structure melted uniformly^28^.

To be sure that metal-coated surface of particles didn't erode during experiment, we examined grains before and after discharge exposure by scanning electron microscopy. No any noticeable changes in the composition and surface properties were detected. At the same time, the original particles had small defects in the coating, which, nevertheless, was not dramatic for the possibility of obtaining a monolayer structure.

**Figure 2:**
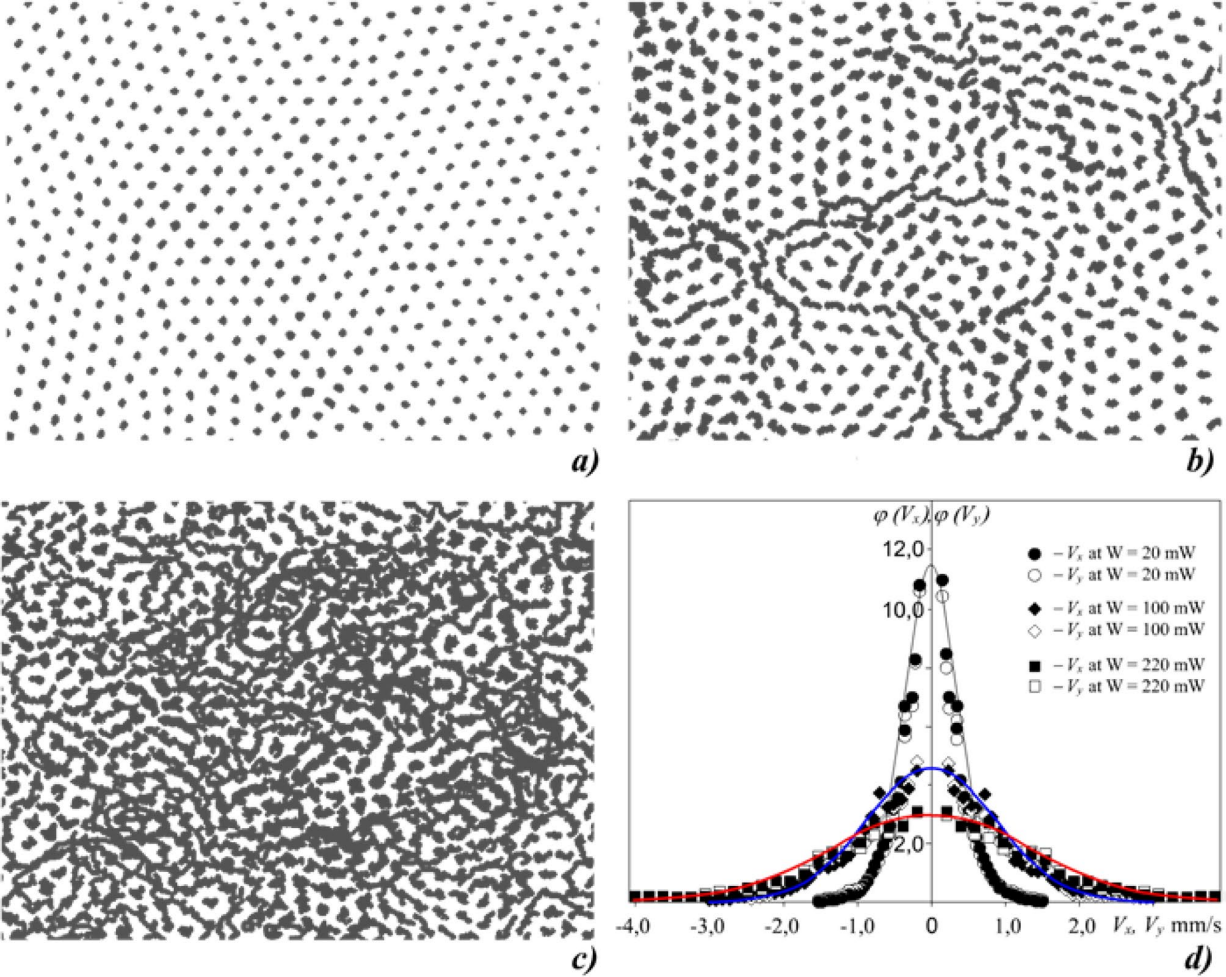
Illustration of typical 1 s particle trajectories in dust monolayer in RF discharge under laser irradiation of power *W*: (**a**) 20 mW, (**b**) 100 mW, (**c**) 220 mW. (**d**) Velocity distribution profiles (symbols) with Maxwell approximation (solid lines): black is for Γ^*^ ~ 600 (W = 20 mW), blue is for Γ^*^ ~ 140 (W = 100 mW) and red is for Γ^*^ ~ 50 (W = 220 mW).

The movements of dust particles in the horizontal plane were recorded by a high-speed video camera with a frame frequency of 50–200 frames/s. The observed dust cloud consisted of a single dust layer with a number of particles up to 10^4^. As the intensity of the laser radiation increased, the kinetic heating of the dust particles occurred, and the structure transferred from the crystalline to the liquid state. At this, to monitor the structure being a monolayer, we installed an additional CCD-camera to record particle movements in vertical direction through the side window of the discharge chamber. The obtained video images were processed using original computer programs, as a result of which the coordinates of the particles, their trajectories (see Fig. 2) and the speed of movements were determined.

The resulting velocity distribution of dust particles corresponded to the Maxwellian distribution function (within the experimental error), thus, for the dust component of the plasma the local equilibrium was observed and we could use term temperature to mean kinetic energy of motion (*V*_T_^2^ = 2* T*/*M*). At this there was a uniform redistribution of the velocities between the degrees of freedom (in a horizontal plane). Velocity variation in a measuring area was random and the errors didn’t exceed 7%. During the time of observation (10 s) a particle kinetic temperature, averaged over all particles in analyzed area, was also within the experimental error (~ 7%). Illustration of typical 1 s particle trajectories together with the velocity distribution profiles for different values of heating laser power is shown in Fig. 2.

From our point of view, there are two main reasons prevented from detection of two-step melting of 2D dusty plasma crystals in previous experiments of other authors: substantial inhomogeneity of melting process, causing temperature gradients and shear flows^40–45^; and a little time for long-range relaxations before direct registration of the structural parameters of the analyzed dust structures^36^.

To achieve the uniformity of the kinetic heating of dusty structure we expanded laser beam by cylindrical lens and then cut off non-homogeneous parts of laser radiation by a diaphragm so that only the central part of widened beam with a uniformity of better than 95% reached dust particles. In addition, metal-coated particles moved chaotically rather than uncoated ones as a response to external force. At such conditions we observed an increase of particle kinetic energy without any evidence of any collective motions in a preferred direction, so that dusty plasma structure melted as a whole.

Before video recording dusty plasma structure has been kept under constant parameters of discharge and power of laser radiation during a time, sufficient for establishing not only stationarity and homogeneity of the system (which is commonly of about several seconds), but also for long-range relaxations within structure after starting laser exposure^36^. (In our experiments it equaled ~ 5–10 min).

Another advantage of our method is the ability to vary precisely the power of laser radiation and thereby change with a small step dust kinetic temperature without loss of heating uniformity so that we obtained a big set of experimental «points» with slightly varied dust parameters and still constant plasma parameters. To sum up, long time for establishing stationarity and homogeneity of the system during experiments together with original precision method of manipulating the kinetic temperature of dust particles allowed us to fulfill conditions needed to «catch» hexatic phase, never observed before in dusty plasmas.

## Methods and results

In order to avoid using of individual characterictics, specific to each experiment, and to make it possible to compare obtained data with numerical results, there is widely used effective coupling parameter Γ^*^ = 1.5(*eZ*)^2^(1 + *κ* + *κ*^2^/2)exp(−*κ*)/(*T*_d_*r*_p_), where *κ *≡ *r*_p_/*λ* is the screening parameter, *r*_p_ is the interparticle distance, *T*_d_ is the kinetic temperature of the particles^49^. This parameter exhibits dimensionless properties for 2D—Yukawa systems, describing extended monolayer dusty plasma structures. Numerical calculations for two-dimensional Yukawa systems show that the physical properties of such systems have two characteristic points of phase transitions^25,26^. The first of these relates to the phase transition "liquid—hexatic phase" and occurs when the effective coupling parameter of Γ^*^ = 98 ± 4; the second point (at Γ^*^ = 154 ± 4) corresponds to the transition from the hexatic phase to the ideal crystal, where the diffusion coefficient of the particles tends to zero. In our experiment we determined the effective coupling parameter Γ^*^ by the first peak of pair correlation function^49^ within the accuracy of 5–10% (depending on Γ^*^).

For the quantitative and qualitative description of the phase state of the system, as a rule, the analysis of pair *g*_2_(*r*) and bond-angular *g*_6_(*r*) correlation functions is used, as well as the dynamics of various topological defects^5–10^. For an ideal hexagonal structure the function *g*_6_(*r*) ≡ 1, while it decreases with distance for other phase states of the system. The asymptotics of dimensionless pair *g*_2_(*r/r*_p_) and bond-angular *g*_6_(*r/r*_p_) correlation functions can be used to analyze the phase state of the system^27,28^. Thus, for two-dimensional non-ideal systems, the spatial decay of peaks of pair correlation functions in an ideal crystal is described by the power law *g*_2_ ∝ (*r*/*r*_*p*_)^-η^ at *η* < 1/3, in the hexatic phase and the liquid, by the exponential dependence *g*_2_ ∝ exp(-*μr/r*_p_) at *μ* = *μ*_*h*_≡ *const* and *μ* > *μ*_*h*_, respectively. The bond-angular correlation functions are characterized by a power asymptotic in the hexatic phase and exponential in the liquid phase.

**Figure 3:**
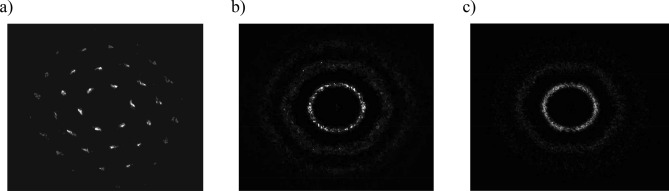
Two-dimensional static structure factor *s*(**k**_*xy*_), calculated for various values of effective coupling parameter Γ^*^: (**a**) Γ^*^ ~ 600; (**b**) Γ^*^ ~ 140; (**c**) Γ^*^ ~ 50.

As it has been shown in our previous studies^25–28^, the value of the function *g*_6_(*r/r*_*p*_) is completely determined by the number of the appearing defects, that is why it is more convenient and reasonable to use normalized bond-angular correlation function in the form of *g*_6_^*^(*r/r*_*p*_) = g_6_(*r/r*_*p*_)/*N*_*6*_, where *N*_*6*_ is a fraction of particles with 6 nearest neighbors (see Fig. 3 in our work^28^). There we presented three sets of clearly resolved curves with different slopes, corresponding to crystal, hexatic, and liquid phases as predicted by KTHNY theory: *g*_6_^*^(*r/r*_*p*_) ~ Const for the range of effective coupling parameter Γ^*^ = 160–220, *g*_6_^*^(*r/r*_*p*_) ~ *r* ^-η^_6_ at η_6_ = 1/5 for Γ^*^ = 110–140, and *g*_6_^*^(*r/r*_*p*_) ~ exp(− *r/ξ*_6_*r*_*p*_) for Γ^*^ = 10–55, respectively.

In this paper we present important information about the phase states of the system under study, which can be obtained from the analysis of the diffraction pattern correlated with the configuration of the particles in this phase. For this purpose, the static structure factor *s*(**k**_*xy*_) was calculated, defined as^50^:

$$s({\mathbf{k}}_{xy} ) = \frac{1}{N}\left\langle {\sum\limits_{n = 1}^{N} {e^{{iq{\mathbf{k}}_{xy} \cdot {\mathbf{r}}_{n} }} } \sum\limits_{m = 1}^{N} {e^{{ - iq{\mathbf{k}}_{xy} \cdot {\mathbf{r}}_{m} }} } } \right\rangle ,$$

where **k**_*xy*_—wave vector, **r**_*n*_ and **r**_*т*_—radius-vectors for *n*-th and *m*-th particle on *xy-*plane, correspondingly. Brackets < > define ensemble and time averaging. Figure 3 shows two-dimensional static structure factor *s*(**k**_*xy*_), calculated for various values of effective coupling parameters Γ^*^, illustrating various diffraction patterns, specific to the solid (a), hexatic (b) and liquid (c) phases of the non-ideal system. It is clearly seen that in the crystalline phase there are clear diffraction maximums corresponding to the hexagonal lattice. With increasing temperature the peaks become blurred up to forming hexagons, indicating the formation of an intermediate state, and then they form concentric circles, typical of the liquid phase.

Analysis of the asymptotic behavior of the correlation functions and diffraction patterns obtained from the calculation of the structural factor allows us to distinguish different phase states of two-dimensional non-ideal systems. However, it becomes less informative when considering asymptotics in the area of phase transitions, where the error in determining various thermodynamic and structural characteristics increases in close proximity to critical point of phase transition. In order to avoid the ambiguity of the analysis associated with fluctuations of spatial parameters and edge effects due to the finite size of the structure and averaging time, as well as to accurately determine the phase transition points, we used the method described in^17^. It is based on the analysis of fluctuations of the bond-angular and translational order parameters by calculating the susceptibilities of the corresponding global order parameters defined by the following formula:

$$\chi_{\alpha L} = L^{2} (\left\langle {\left| {\Psi_{\alpha }^{2} } \right|} \right\rangle - \left\langle {\left| {\Psi_{\alpha } } \right|} \right\rangle^{2} ).$$

Here *L*—size of the system under consideration, index α = 6, T defines bond-angular and translational order, respectively, and $\Psi_{\alpha } = \left| {\frac{1}{N}\sum\limits_{j = 1}^{N} {\psi_{\alpha ,j} } } \right|$—global order parameter, determined as the local order parameter *ψ*_α,j_ averaged by all *N* particles *j*, taken in box of *L* x *L* size, given in average interparticle distances. Local order parameters for the particle *j* at position **r**_*j*_ = (*x*_*j*_, *y*_*j*_) are defined as:

- for bond-angular order $\psi_{6,j} = \frac{1}{{N_{j} }}\sum\nolimits_{j = 1}^{{N_{j} }} {\exp (6i\theta_{j,k} )}$, where the sum on *j* is over all nearest neighbors *N*_*j*_ of the *j*^*th*^ particle, and an angle $\theta_{jk}$ is formed by a bond, connected the *k*^*th*^ and the *j*^*th*^ particles, and a fixed axis;
- for translational order $\psi_{T,j} = \exp (i{\mathbf{Gr}}_{j} )$, where **G**—primary vector of the reciprocal lattice, determined by the peak of the two-dimensional structure factor *s*(**k**_*xy*_) for each temperature value.

For the liquid and hexatic phases, it is often difficult to determine magnitude of **G**. In this case, for the initial evaluation we used the value **G**, obtained for crystal phase, and then maximized the value of *ψ*_*T*_ by varying the vector **G** in the neighborhood of the initial estimate found from *s*(**k**_*xy*_). Resulting value **G** was assumed optimal for a specific temperature value and was used in subsequent calculations of the global and local translational order parameter and the corresponding susceptibilities.

**Figure 4:**
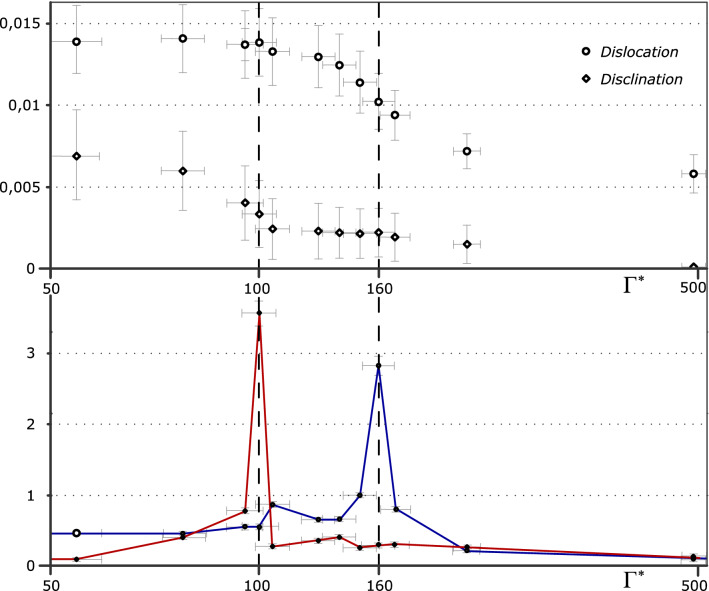
(top) Free dislocations (circles) and free disclinations (diamonds) relative fraction; (bottom) The translational, *χ*_*T*_, and orientational, *χ*_6_, susceptibilities as a function of coupling parameter Γ^*^. The divergence of *χ*_*T*_ and *χ*_6_ clearly indicates two transition points at Γ^*^ ~ 100 and Γ^*^ ~ 160 (vertical dashed lines).

To calculate accurately the susceptibilities *χ*, it is necessary to collect sufficient statistics over time (in our case, the calculations were averaged over 2000 frames). To eliminate effects associated with the finite size of the structure, the calculation of *χ*_L_ was carried out in subboxes of various sizes *L*, and then was extrapolated in the thermodynamic limit to *χ*_∞_. Starting from *L* = 30–40 interparticle distances (i.e. a region with 1500–2000 particles), the value of the parameter *χ*_L_ didn’t largely change (within 5% error), and tended to *χ*_∞_, i.e. *χ*_L_ → *χ*_∞_≡*χ*. Figure 4 (bottom) shows a plot of the translational, *χ*_T_, and orientational susceptibilities *χ*_6_, in dependence of the effective coupling parameter Γ^*^.

One can see the jumps of the functions *χ*_T_ and *χ*_6_, marked by vertical dashed lines, which clearly indicate two points of phase transitions at Г^*^ ~ 100 and Г^*^ ~ 160, respectively. The susceptibilities calculated for regions with a smaller number of particles (of about 400–500) also had smaller, but still clearly detectable jumps at the same points for value Г^*^. Thus, this method for analyzing the susceptibility of order parameters showed good persistence to edge effects and the possibility of its use not only for extended dusty plasma structures, but also for a relatively small number of particles, in contrast to the method based on the calculation and analysis of correlation functions and structure factor.

**Figure 5:**
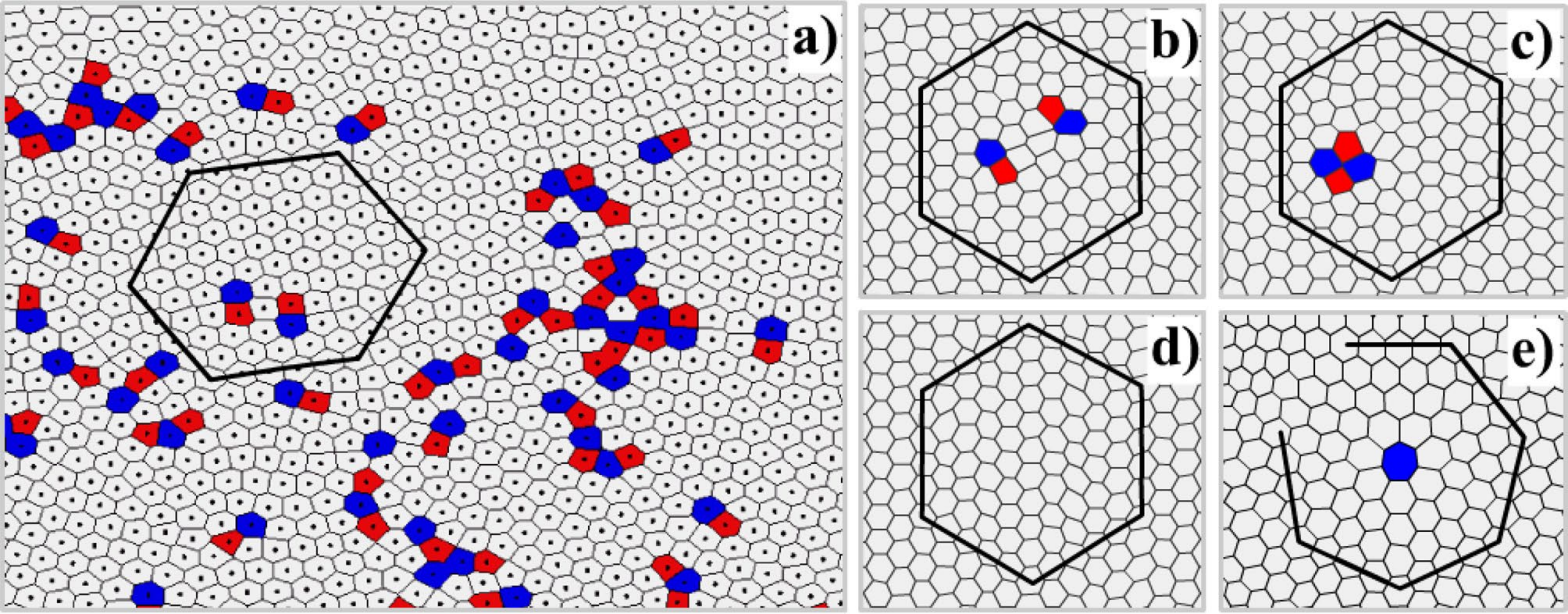
Illustration of (**a**) the Voronoi diagram for the hexatic phase at Γ^*^ ~ 140. Dots indicate particles’ position. The red and blue Voronoi cells are marked for 5- and 7-folded particles, respectively; gray cells represent nondefect particles with 6 nearest neighbors. Subplot illustrates Burgers path with zero vector for different cases: (**b**) free dislocations with opposite Burgers vectors located on one lattice row, (**c**) dislocation pair (**d**) no defects. All three cases are interchangeable in time. (**e**) 7-folded disclination with non-zero Burgers vector.

Let’s take a look more closely on the analysis of defects occurring in a two-dimensional nonideal dusty plasma structure. For a hexagonal lattice, the most common defects are disclinations—isolated defects with 5 or 7 nearest neighbors, dislocations—5–7 pairs of disclinations, and dislocation pairs—5–7–5–7 quadruple disclinations. It is convenient to visualize the picture of emerging defects, as well as their evolution over time, using the Voronoi diagram. An illustration of the construction of the Voronoi diagram for an experimentally obtained dusty plasma structure with Γ^*^ ~ 140 is shown at Fig. 5.

According to BKTHNY—theory, unbinding of dislocation pairs to free dislocation (i.e. isolated 5–7 pairs of disclinations) cause “solid-to-hexatic” phase transition, and unbinding of dislocations to free disclinations (i.e. isolated 5- or 7-folded particles) cause “hexatic-to-liquid” phase transition. The dislocation acts as an additional row of particles, which gives a nonzero Burgers vector^3,11^ and effectively destroys the translational order in the system, while keeping the orientational order (see Fig. 5a–d). Two oppositely oriented dislocations form a pair with a zero Burgers vector, which does not violate translational and orientational symmetry. Therefore, dislocation pairs can be formed due to thermal excitation even at low temperatures in the crystal. As for disclinations, they strongly violate both translational and orientational symmetry (see Fig. 5e), that’s why they appear at higher temperatures and almost absent in crystal.

We experimentally measured the relative fractions of free disclinations and dislocations (i.e. surrounded by 6-folded particles) occurred in the system, depending on the effective coupling parameter Γ^*^ (see top of Fig. 4). The figure shows that in the crystalline phase the concentration of free defects is vanishingly small. With increasing temperature in the system (and therefore decreasing the value of the effective coupling parameter Γ^*^), one can see a gradual increase in the concentration of free dislocations at Γ^*^ ~ 170, and then growth in the number of free disclinations at Γ^*^ ~ 110 begins.

Whereas this graph shows qualitatively that the melting process is connected with increase of defects, it doesn’t accurately determine the position of critical points. The problem is that the statistics inevitably includes dislocations that are not quite “free”, i.e. have a pair on the same line of the lattice, and thus have a zero Burgers vector (see Fig. 5b–d). Such dislocations rapidly evolve in time. In particular they present “hopping”, or collapsing, or forming a dislocation pair, or “running” along one line of the lattice for some distance from each other. Another problem is the high sensitivity of the calculation to systematic errors, since defects tend to form large clusters that can correspond to different numbers of free dislocations or disclinations. For example, a 6-mer 5–7-5–7-5–7 can be counted as a single dislocation added to a dislocation pair, or as three dislocations with the codirected Burgers vectors. This may explain the fact that the growth of dislocations on the graph occurs a little earlier than the melting in the system. It can be assumed that, before melting some seed concentration of clusters of nonfree defects needed for the formation of stable free dislocations should be accumulated in the system. The same behavior was observed in colloids^17^, but had never been seen in dusty plasma before. We should note, that we didn’t observe the clear process of “unbinding” of free dislocations to separate free disclinations, considered in BKTHNY-theory. The most common pattern was developing of dislocations to agglomerations, consisting of several 5- and 7-folded particles. We presume that it happens because such formation of bigger defect on the base of “seed” dislocation costs less energy compared to creation of free disclination.

Finally, let’s consider the core energy of free dislocations, *E*_*c*_, which is an essential parameter of the 2D system. According to the *Grain-Boundary-Induced melting (GBI-)* theory, first-order phase transition (by the formation of a polycrystalline structure) preempts KTHNY-scenario under the condition *Ec* < 2*.*84 *k*_*B*_*T*. One can observe such situation, for example, in^43^. The value of *E*_*c*_ can be obtained from the Boltzmann distribution of free dislocations:

$$\frac{\eta }{1 - \eta } \propto \exp ( - {{E_{{\text{c}}} } \mathord{\left/ {\vphantom {{E_{{\text{c}}} } {k_{{\text{B}}} T}}} \right. \kern-\nulldelimiterspace} {k_{{\text{B}}} T}}),$$

where *η−* free dislocation density. However the complex structure of the occurring defects makes the determination of value *η* rather problematic, as it was mentioned above. The core energy can be roughly estimated by measuring the density of particles with the number of nearest neighbors other than six (1 − *N*_6_), i.e. by taking into account all defects. Such a method was used earlier in^51^, but at the same time it does not take into account the interaction of defects and leads to an overestimation of the number of dislocations, and hence an underestimation of the value of *E*_*c*_:

$$\frac{{1 - N_{6} }}{{N_{6} }} \propto \exp ( - {{E_{{\text{c}}} } \mathord{\left/ {\vphantom {{E_{{\text{c}}} } {k_{{\text{B}}} T}}} \right. \kern-\nulldelimiterspace} {k_{{\text{B}}} T}})$$

**Figure 6:**
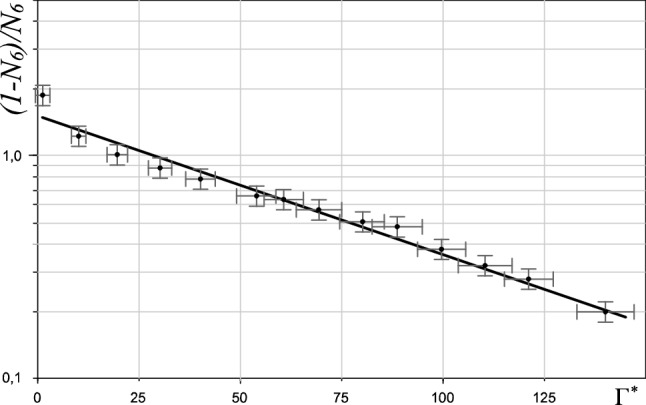
Log–norm plot of the ratio of the defect fractions to the nondefect fraction, (1 − *N*_6_)/*N*_6_ as a function of effective coupling parameter Γ^*^.

The plot of the dependence of the magnitude ${{(1 - N_{6} )} \mathord{\left/ {\vphantom {{(1 - N_{6} )} {N_{6} }}} \right. \kern-\nulldelimiterspace} {N_{6} }}$ on the effective coupling parameter Γ^*^, which in this case acts as the equivalent of the inverse temperature 1/*T* (i.e. Γ* ~ 1/*T*) is presented in Fig. 6.

As one can see at Fig. 6, the experimental points fit well on a straight line, the angle of which yielded us estimation of the core energy *E*_*c*_: *E*_*c*_ = 3.1 ± 0.1*k*_*B*_*T*. The real value of *E*_*c*_ is higher, since the magnitude obtained from the plot is the lower-bound estimate. As can be seen from the obtained estimation, the measured value of *E*_*c*_ is obviously above the threshold value of 2.84*k*_*B*_*T* predicted in^12^, which is yet more proof that the melting process of two-dimensional dusty plasma structures follows the BKT—scenario. Presented analysis shows that melting scenario is highly sensitive to the way applied to transfer 2D system from one phase state to another.

## Conclusions

To summarize, we consider a method of kinetic heating of dusty plasma structures under constant plasma parameters of the discharge, based on the influence of enlarged homogeneous laser beam on entire dusty plasma system, resulting in melting of 2D dusty plasma crystal. Quantitative and qualitative analysis of static structure factor, local and global parameters of translational and bond-angular order parameters, temperature dependence of various types of defects, as well as estimating core energy of free dislocations and construction of Voronoi diagrams show clear evidence of two-step process of melting from the crystal to the liquid phase with formation of intermediate hexatic phase, predicted by BKT-theory. Using translational and orientational susceptibilities we accurately identified «solid-to-hexatic» and «hexatic-to-liquid» phase transition points. Long time for establishing stationarity and homogeneity of the system during experiments together with precision method of manipulating the temperature of dust particles allowed us to observe the clear evidence of hexatic phase never seen before in laboratory dusty plasma systems.
